# Specific and non-specific binding of a tracer for the translocator-specific protein in schizophrenia: an [11C]-PBR28 blocking study

**DOI:** 10.1007/s00259-021-05327-x

**Published:** 2021-04-06

**Authors:** Tiago Reis Marques, Mattia Veronese, David R. Owen, Eugenii A. Rabiner, Graham E. Searle, Oliver D. Howes

**Affiliations:** 1 Psychiatric Imaging Group, MRC London Institute of Medical Sciences (LMS), Hammersmith Hospital, Imperial College London, London, UK; 2 Psychiatric Imaging Group, Institute of Clinical Sciences (ICS), Faculty of Medicine, Imperial College London, London, UK; 3 Department of Psychosis Studies, Institute of Psychiatry, Psychology and Neuroscience, Kings College London, London, UK; 4 Centre for Neuroimaging Sciences, Institute of Psychiatry, King’s College London, London, UK; 5 Division of Brain Sciences, Department of Medicine, Imperial College, London, UK; 6 Invicro, London, UK

**Keywords:** TSPO, Schizophrenia, Microglia, XBD173

## Abstract

**Objective:**

The mitochondrial 18-kDa translocator protein (TSPO) is expressed by activated microglia and positron emission tomography enables the measurement of TSPO levels in the brain. Findings in schizophrenia have shown to vary depending on the outcome measure used and this discrepancy in TSPO results could be explained by lower non-displaceable binding (*V*_ND_) in schizophrenia, which could obscure increases in specific binding. In this study, we have used the TSPO ligand XBD173 to block the TSPO radioligand [^11^C]-PBR28 and used an occupancy plot to quantify *V*_ND_ in patients with schizophrenia.

**Methods:**

A total of 7 patients with a diagnosis of schizophrenia were recruited for this study. Each patient received two separate PET scans with [^11^C]PBR28, one at baseline and one after the administration of the TSPO ligand XBD173. All patients were high-affinity binders (HABs) for the TSPO gene. We used an occupancy plot to quantify the non-displaceable component (*V*_ND_) using 2TCM kinetic estimates with and without vascular correction. Finally we computed the *V*_ND_ at a single subject level using the SIME method.

**Results:**

All patients showed a global and generalized reduction in [^11^C]PBR28 uptake after the administration of XBD173. Constraining the *V*_ND_ to be equal for all patients, the population *V*_ND_ was estimated to be 1.99 mL/cm^3^ (95% CI 1.90 to 2.08). When we used vascular correction, the fractional TSPO occupancy remained similar.

**Conclusions:**

In schizophrenia patients, a substantial component of the [^11^C]PBR28 signal represents specific binding to TSPO. Furthermore, the *V*_ND_ in patients with schizophrenia is similar to that previously reported in healthy controls. These results suggest that changes in non-specific binding between schizophrenia patients and healthy controls do not account for discrepant PET findings in this disorder.

**Supplementary Information:**

The online version contains supplementary material available at 10.1007/s00259-021-05327-x.

## Introduction

Schizophrenia is the result of a complex interplay between multiple pathophysiological mechanisms [18]. Recent studies have produced converging evidence supporting a role of inflammation and immune response as an important contributor to the pathogenesis of this disorder [17]. In the central nervous system, microglia cells play a key role in the response to an inflammatory stimulus, changing from a quiescent (resting) state to an activated state and releasing pro-inflammatory cytokines. When microglia are activated, they increase the expression of the mitochondrial 18-kDa translocator protein (TSPO) [6]. By using radioligands targeting this protein, positron emission tomography (PET) enables the measurement of TSPO levels in the brain. Multiple studies have used PET tracers for TSPO to investigate this marker in schizophrenia-spectrum disorders ([2, 4, 11, 15, 41, 10, 16, 37, 5, 9, 29, 32, 40, 41]). However, findings have varied depending on the outcome measure used, with a recent meta-analysis showing that TSPO PET tracer binding is significantly elevated in patients with schizophrenia relative to controls when binding potential (BP) is used as an outcome measure, but when the tracer volume of distribution (*V*_T_) is used as the outcome measure, no significant differences are seen between patients with schizophrenia and healthy controls [26]. Binding potential uses a reference region to account for binding of the tracer to other brain constiuents other than TSPO, often termed non-displaceable binding, while *V*_T_ represents both specific binding to TSPO and non-displaceable binding by the tracer [20]. Non-specific binding refers to the radioligand non-specifically bound to molecules other than the target of interest and free radioligand in the tissue ([20]; Rosso et al., 2008; Ogden et al., 2014). Thus, the discrepant TSPO results could be explained by lower non-displaceable binding in schizophrenia, which could obscure increases in specific binding, accounting for the lack of differences reported in many of the studies, and a small decrease in *V*_T_ on a pooled analysis [32]. However, it is not known if non-displaceable binding is altered in schizophrenia. Non-displaceable binding (*V*_ND_) can be estimated through pharmacological blockade using a drug that blocks tracer binding to the protein of interest. Importantly, this approach is considered the “gold standard” for the in vivo estimatation of *V*_ND_. A previous study in healthy controls used a drug that selectively binds to the TSPO (XBD173) to block the specific binding signal of [^11^C]PBR28 and calculate the *V*_ND_ [31]. By using an occupancy plot, the *V*_ND_ at a population level was calculated as being 1.98 mL/cm^3^. Finally, a recent study used the same method to calculate the *V*_ND_ of [11C]PBR28 in multiple sclerosis as being 3.81 mL/cm^3^, which suggests *V*_ND_ may be altered in pathological states (Sridharan et al., 2019). As *V*_ND_ is unknown in schizophrenia, we performed a PET imaging study using pharmacological blockage with XBD173 to calculate, for the first time, the *V*_ND_ in patients with schizophrenia.

## Material and methods

### Participants and experimental protocol

The study protocol was approved by the National Research Ethics Service (NRES) and permission to administer radioactive substances was granted by the Administration of Radioactive Substances Advisory Committee (ARSCAC), UK. All participants gave written informed consent to participate after receiving a full description of the study. In total, seven patients with a diagnosis of schizophrenia were recruited from the South London and Maudsley Foundation NHS Trust. Out of the 7 participants, baseline, but not blocking data, were included from 6 in a previous report (Bloomfiled et al., 2016). Diagnosis was ascertained through clinical interview and reviewing medical records, and all patients met DSM-5 criteria for schizophrenia according to the Structured Clinical Interview of DSM-5-TR Axis I Disorders (SCID). Subjects were then screened based on the following exclusion criteria: exposure to any medication except antipsychotics, including anti-inflammatory and benzodiazepine medications, in the last month; history of substance abuse/dependence as determined by the Structured Clinical Interview of DSM-5-TR Axis I Disorders (SCID) [13]; history of head injury resulting in unconsciousness, or any physical medical condition associated with inflammation; low-affinity binder for the TSPO gene; significant prior exposure to radiation; pregnancy or breast feeding. Patients were genotyped for the TSPO genetic polymorphism and all subjects were high affinity binders (HABs) for this polymorphism.

Each patient received two separate PET scans with [^11^C]PBR28, one at baseline and one after XBD173 administration. As circadian rhythms may impact TSPO measurement [3, 7], PET scans were conducted at similar time of the day (mean injection time: 14:32; earliest injection: 10:46; latest injection: 15:56) in order to reduce intra and inter-subject variability. At the time of PET scanning, all patients were stabilized on antipsychotic treatment. At both baseline and follow-up visits, subjects had a physical, psychiatric, and neurological examination and no participant had a history of other neurological or psychiatric disorders other than schizophrenia. Clinical measures were recorded at both PET scans, including physical examination and medication history. Drug screens were done on the days of the scans to exclude the use of psychoactive drugs. Psychotic symptoms were evaluated using the Positive and Negative Syndrome Scale (PANSS) [21].

### XBD173 (emapunil) administration

XBD173 is a selective TSPO agonist, binding to this site with nanomolar affinity [31]. All seven participants received a standard oral dose of 90 mg of XBD173 corresponding to 0.98 ± 0.16 mg/kg (mean±SD) based on the patient weights. This dose has been previously tested in healthy controls and was shown to be safe, well tolerated with no reported side effects [31]. This dose aimed to reach between 66 and 77% of TSPO brain occupancy and was given 2 h prior to radiotracer injection, which was timed to coincide with the anticipated peak concentration of XBD173 in blood [31].

### Neuroimaging evaluation

#### Image acquisition

Participants were instructed to refrain from caffeine, tobacco, and alcohol for at least 12 h before scanning. All participants were scanned twice on a Siemens Biograph TruePoint PET/CT scanner (Erlangen, Germany) following the injection of an intravenous bolus [^11^C]PBR28. Dynamic emission data were acquired continuously for 90 min following the injection of [^11^C]PBR28. PET data were acquired in three-dimensional mode and binned into 26 frames (durations: 8 × 15 s, 3 × 1 min, 5 × 2 min, 5 × 5 min, 5 × 10 min). Images were reconstructed using filtered back projection and corrected for attenuation and scatter. During PET acquisition, arterial blood data were collected via the radial artery using a combined automatic-manual approach. A continuous sampling system (ABSS Allogg, Mariefred, Sweden) was employed to measure whole blood activity for first 15 min of each scan at the rate of one sample per second. Discrete blood samples were manually withdrawn at 5, 10, 15, 20, 25, 30, 40, 50, 60, 70, 80, and 90 min, centrifuged, and used to determine the plasma over blood activity ratio (POB). Samples at 5, 10, and 15 min were used to calibrate the two sampling modalities. Samples taken at 5, 10, 20, 30, 50, 70, and 90 min were also analyzed using HPLC to calculate the plasma fraction of tracer free of metabolites (PPf). Radiometabolite analysis of [^11^C]PBR28 blood data was done as described by Owen et al. [31], consistent with previous studies [2]. Parent plasma fraction (PPf) and plasma over blood (POB) were fitted with an extended Hill model [12]. Whole blood TACs were fitted using a multi-exponential model [39]. For each scan, a time delay was fitted and applied to the input functions (both parent and whole blood TACs) to account for temporal delay between blood sample measurement and the target tissue data. MRI scans were acquired with a 32-channel head coil on a Siemens Magnetom Verio, 3-T MRI scanner and included a T1-weighted magnetization prepared rapid gradient echo sequence (MPRAGE; time repetition (TR) = 2300 ms, time echo (TE) = 2.98 ms, flip angle of 9°, time to inversion (TI) = 900 ms, matrix = 240 × 256) for co-registration with the PET images. All sequences used a 1 mm^3^ voxel size, anteroposterior phase encoding direction, and a symmetric echo.

### [^11^C]PBR28 PET data processing

#### Data analysis

Each subject’s structural MRI image underwent brain extraction and gray matter segmentation using Statistical Parametric Mapping (SPM) 8 (http://www.fil.ion.ucl.ac.uk/spm). A neuroanatomical atlas (CIC atlas 2.0) was co-registered to each subject’s MRI scan and PET image using a combination of SPM 8 and FSL (http://www.fsl.fmrib.ox.ac.uk/fsl) functions, implemented in the MIAKAT™ software package (http://www.imanova.co.uk).

Region of interest (ROI) definition included the occipital lobe, temporal lobe, frontal lobe, parietal lobe, insular cortex, cingulate cortex, amygdala, hippocampus, thalamus, striatum, and cerebellum. Whole brain and gray and white matter were also included in analysis. Dynamic PET images were linearly registered to each subject’s MRI scan and corrected for subject motion using non-attenuation-corrected images, as they include greater scalp signal, which improves re-alignment compared to attenuation-corrected images. Frames were realigned to a single “reference” space identified by the PET frame with the highest activity. The transformation parameters were then applied to the corresponding attenuation-corrected PET frames, creating a movement-corrected dynamic image for analysis. ROIs were applied to the dynamic PET data to derive regional time-activity curves (TACs). To investigate the effect of XBD173 on [^11^C]PBR28 tissue uptake, standardized uptake values (SUV), using injected dose and patient weight as normalization factors, were computed 90 min after tracer injection for each PET scan.

#### Kinetic modeling

For consistency with previous blocking studies with TSPO PET tracers [19, 22, 31], quantification of [^11^C]PBR28 tissue distribution was performed using a standard 2TCM, using the metabolite corrected plasma input function. The 2TCM model was solved using the weighted non-linear least square estimator (WNLLS), as implemented in Matlab2012b (The Mathworks Inc., Natick, MA). The total distribution volume (*V*_T_) was then determined for each one of the ROI examined. Regions with *V*_T_ estimates higher than 10 mL/cm^3^ or with unreliable precision (CV > 50%) were excluded from the analysis as non-physiological based on previous analysis [34]. In addition, we have tested the 2TCM1K model to test whether the inclusion of a vascular compartment would predict a different percentage of specific binding than standard 2TCM. Finally, we compute the *V*_ND_ at a single subject levels using the SIME method [38].

#### Quantification of the non-displaceable component (*V*_ND_)

The occupancy plot [8], a revisited version of the original Lassen plot for the calculation of the fractional receptor availability in molecular imaging studies [24], assumes that the *V*_ND_ of the radioligand is unchanged by the administration of the blocking drug, and that the fractional occupancy of the target by the blocking drug is the same across all ROI. With these assumptions, it follows that:

$$ {V}_{\mathrm{T}}\ \mathrm{Baseline}-{V}_{\mathrm{T}}\ \mathrm{Blocking}=\mathrm{Occupancy}\times \left({V}_{\mathrm{T}}\ \mathrm{Baseline}\hbox{--} {V}_{\mathrm{ND}}\right) $$

Therefore, by plotting (*V*_T_ Baseline - *V*_T_ Blocking) versus *V*_T_ Baseline, it is possible to obtain a measure of both target occupancy (the slope) and the *V*_ND_ (the *x*-intercept). Baseline and post-XBD173 *V*_T_ data for each one of the ROI in each of subjects were fitted to the occupancy plot. *V*_ND_ values were then calculated individually for each subject. Consistent with previous literature ([31]; Sridharan et al., 2019), to increase noise robustness, all subjects were constrained to the same *V*_ND_ value. This approach allowed us to compare differences in *V*_ND_ between healthy population and a clinical one.

#### Implementation SIME method

The SIME method requires to define a priori a grid of possible *V*_ND_ values. Then, for each element of the grid, a 2TCM is fitted to all TACs by constraining *K*_1_/*k*_2_ to be equal to chosen *V*_ND_ value in all the ROIs. The residual sum of squares is then computed for all ROIs and frames, and the *V*_ND_ value that yields the lowest residual sum of squares is selected as the estimate of a brain-wide *V*_ND_.

SIME method was implemented consistently with previous applications of the method to 11C-PBR28 PET imaging ([33]; Schain et al., 2019), by using public available code (https://github.com/martinschain/SIME). Only baseline PET scans were considered. The *V*_ND_ grid was selected from 0.01 to 5, with steps of 0.01. Weights and ROIs were kept consistent with the kinetic modeling and occupancy plot analysis.

#### Statistical analysis

Statistical analyses were conducted in SPSS version 19 (www.spss.com). Paired *t*-tests as well as repeated measured ANOVAs (rmANOVA) were used to assess differences between [^11^C]PBR28 PET scans at baseline and after XBD173 administration.

## Results

### Demographic and clinical characteristics of study participants

A total of 7 patients with schizophrenia were included in this study. All patients were male, the mean age of participants at the time of baseline scan was 41.8 ± 14.3 years, and the interval between scans was 743.2 ± 335.2 days. At the time of PET scanning, 6 patients were stabilized and receiving treatment with standard antipsychotics while one patient was unmedicated. Between baseline and follow-up scan, there were minimal changes in antipsychotic medications in 4 patients out of 7, while in two cases, the antipsychotic treatment was changed: case 1 from Amisulpride 200 mg/day to Olanzapine 10 mg/day; case 2 from Olanzapine 10 mg/day to Risperidone 3 mg/day. One subject remained unmedicated at both PET scans (see Table 1).

**Table 1:** Summary of participant demographics, medication, and radioactivity status at baseline and after XBD173 administration

Participant	Interval between scans (days)	Antipsychotic medication	
		Baseline	Follow-up after XBD173 administration
A	954	Clozapine 900 mg + aripiprazol 5 mg	Clozapine 900 mg
B	943	Clozapine 500 mg	Clozapine 500 mg
C	727	Olanzapine 20 mg	Olanzapine 15 mg
D	790	Clozapine 350 mg	Clozapine 300 mg
E	882	Amisulpride 200 mg	Olanzapine 10 mg
F	6	Unmedicated	Unmedicated
G	901	Olanzapine 10 mg	Risperidone 3 mg

There were no significant differences in the injected doses between baseline [^11^C]PBR28 scans (mean baseline radioactivity: 321 ± 32 (SD) MBq) and follow-up XBD173 blocking scans (mean post-XBD173 radioactivity: 302 ± 75 (SD) MBq, (*p* = 0.58)). There was no difference in injected mass (*p* = 0.36) at follow-up (mean post-XBD173 injected mass: 4.1 ± 2.1 (SD) μg) when compared to baseline (mean baseline injected mass: 2.2 ± 0.6 (SD) μg). Similarly, there was a lower (*p* = 0.23) specific activity at baseline (mean baseline-specific activity: 53.7 ± 13.7 (SD) GBq/μmol) when compared to follow-up (mean post-XBD173-specific activity: 32.7 ± 14.6 (SD) GBq/μmol). There were no reported side effects after the administration of XBD173.

### Estimation of non-displaceable volume of distribution

**Fig. 1:**
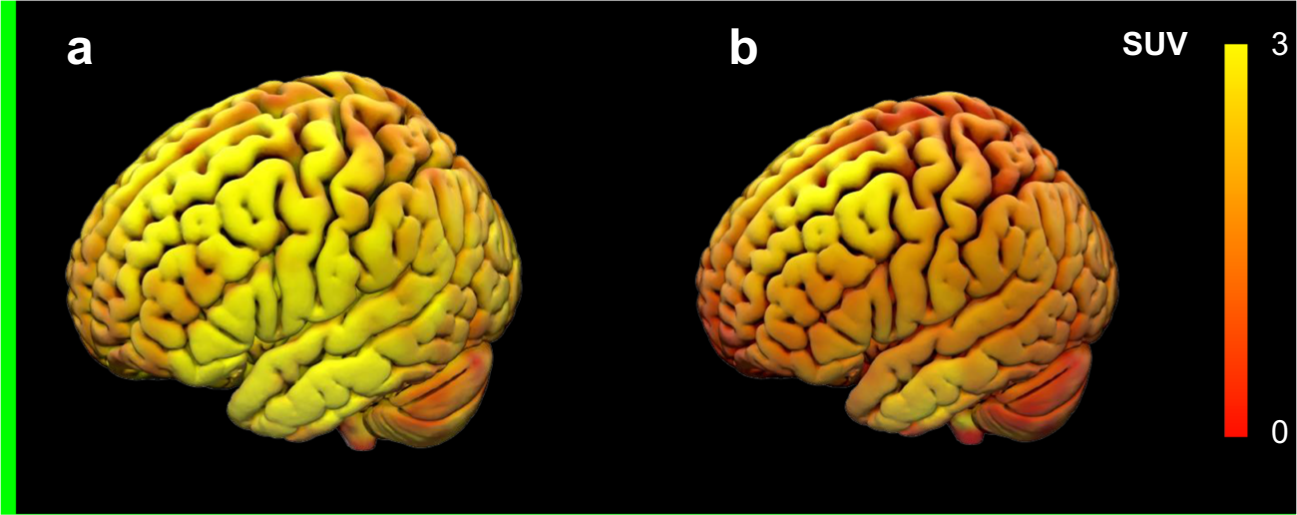
[11C]PBR28 brain PET tissue uptake (quantified with SUV) before (A) and after XBD173 administration (B). Images taken from scans of the same representative patient to show the effect of XBD173 on tracer uptake across the brain

There was a global and generalized reduction in [^11^C]PBR28 uptake after the administration of XBD173 in all subjects (Fig. 1). This reduction was observed in all ROIs analyzed including the cerebellum. Even when the tracer-specific activity was used as a covariate, these reductions remained significant (*p* = 0.02), with no interaction between specific activity and standard uptake value (SUV) (*p* = 0.97). The same pattern of global reduction was present in the *V*_T_ estimates for both standard 2TMC and 2TCM with vascular correction (Table 2) and was confirmed by parametric mapping analysis (Supplementary Material). With 2TCM, all the subjects displayed a measurable occupancy of the TSPO following blockade with XBD173 about 56% ± 12% for whole brain, %54 ± 7% in white matter and %53 ± 18% in gray matter. Similarly, 2TCM1K kinetic analysis led to a fractional occupancy of the TSPO following blockade with XBD173 about 60% ± 24% for whole brain, %49 ± 20% in white matter, and %67 ± 20% in gray matter. These percentage *V*_T_ variations were consistent between the two models (two-tail paired *t*-test *p* > 0.1) although the tracer uptake was differentially associated with different model compartments. For a full analysis of micro- and macro-parameters obtained with 2TCM and 2TCM1K, we refer the interested reader to Veronese et al. [43].

**Table 2:** Volumes of distribution (*V*_T_) in different brain regions at baseline and after XBD173 administration

ROI	Standard 2TCM			2TCM1K		
	Baseline *V*_T_ (mL/cm^3^) Mean ± SD	Post-XBD173 *V*_T_ (mL/cm^3^) Mean ± SD	(*V*_T_) change* (%)	Baseline *V*_T_ (mL/cm^3^) Mean ± SD	Post-XBD173 *V*_T_ (mL/cm^3^) Mean ± SD	(*V*_T_) change* (%)
Whole brain	4.72 ± 1.18	2.16 ± 1.05	54%	2.31 ± 0.63	0.70 ± 0.26	70%
White matter	4.30 ± 0.95	1.97 ± 0.58	54%	1.34 ± 0.38	0.62 ± 0.10	54%
Gray matter	4.95 ± 1.26	2.45 ± 1.62	50%	2.64 ± 0.71	0.76 ± 0.34	71%
Occipital lobe	4.76 ± 1.26	2.91 ± 2.36	39%	2.56 ± 0.73	0.70 ± 0.31	73%
Temporal lobe	4.82 ± 1.16	2.06 ± 0.85	57%	2.43 ± 0.75	0.73 ± 0.29	70%
Frontal lobe	4.81 ± 1.21	2.43 ± 1.57	49%	2.49 ± 0.65	0.73 ± 0.30	71%
Parietal lobe	4.64 ± 1.18	2.91 ± 2.65	37%	2.42 ± 0.67	0.71 ± 0.31	71%
Amygdala	6.67 ± 2.09	1.62 ± 0.53	76%	2.49 ± 1.55	1.14 ± 0.22	54%
Hippocampus	5.25 ± 1.23	1.88 ± 0.51	64%	2.59 ± 1.07	1.04 ± 0.35	60%
Thalamus	5.70 ± 1.28	1.79 ± 0.45	69%	2.45 ± 0.78	0.90 ± 0.29	63%
Striatum	4.83 ± 1.01	1.86 ± 0.60	61%	2.62 ± 1.03	0.74 ± 0.18	72%
Cerebellum	4.87 ± 1.25	2.27 ± 1.44	53%	2.71 ± 0.65	0.78 ± 0.34	71%

*V_T_ change is computed as the relative difference between mean baseline *V*_T_ and mean post-XBD173 *V*_T_

**Fig. 2:**
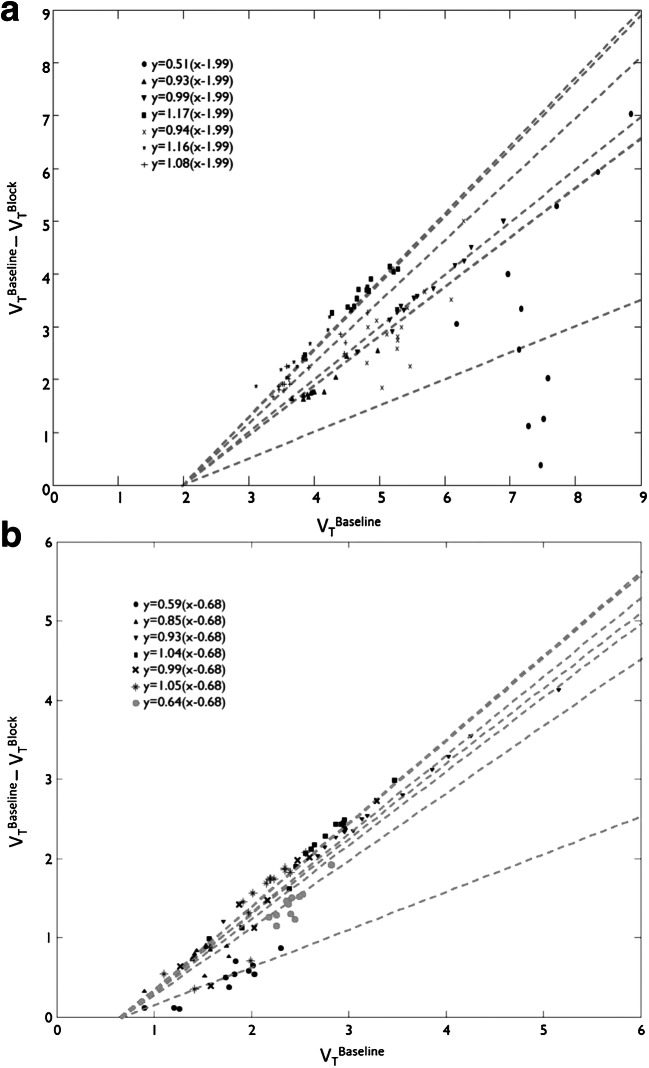
Occupancy plot to determine group mean [^11^C]PBR28 *V*_ND_ with 2TCM (A) and 2TCM1K (B). Each point represents an individual subject ROI value. *V*_ND_ is constrained to be equal for all patients (*N* = 7, all subjects included). Note that the first subject on the list (ID = #305, full circles) is the one that has been identified as the outlier in the individual *V*_ND_ analysis

The regional volumes *V*_T_ were then used in an occupancy plot to calculate the occupancies (occupancy*)* and non-displaceable volume (*V*_ND_). Individual *V*_ND_ values ranged from 1.50 to 5.55 with a group average of 2.59 (*N* = 7, 95% CI: 1.58 to 3.59) and between subject variability of 49%. One subject with very high baseline *V*_T_ values (38% ± 3% greater than the average of the remaining 6 subjects) was mainly responsible for this high variability. When this subject was removed from the analysis, the *V*_ND_ group average was 2.17 (*N* = 6, 95% CI: 1.67 to 2.66) and the between subject variability was 29%. Constraining the *V*_ND_ to be equal for all patients (no outlier removed), the population *V*_ND_ was estimated to be 1.99 mL/cm^3^ (*N* = 7, 95% CI: 1.90 to 2.08) with occupancy of 97% ± 22% (Fig. 2A).

When we used 2TCM1K individual *V*_ND_ estimates calculated with the occupancy plot ranged from 0.14 to 1.03 with a group average of 0.47 (*N* = 7, 95% CI: 0.17 to 0.78), while the *V*_ND_ constrained to be equal for all the patients (*N* = 7, all subjects included) was 0.68 corresponding to a mean occupancy of 92% ± 15% (Fig. 2B). Despite the difference of *V*_T_ estimates, the two models (2TCM and 2TCM1K) returned a similar fraction of specific binding (*V*_S_/*V*_T_), corresponding to 58% ± 10% for 2TCM and to 68% ± 13% for 2TCM1K (*V*_ND_ fixed to population values).

### Simultaneous estimatation vs blocking study

When we used SIME to estimate non-displaceable binding, individual *V*_ND_ estimates (mean±sd: 1.16 ± 0.28 mL/cm^3^) were significantly different than the corresponding one obtained with Lassen plot (mean±sd: 2.65 ± 1.64 mL/cm^3^; paired *t*-test *p*:0.045). On average, the SIME *V*_ND_ estimates were 45% ± 22% lower than those obtained from the blocking studies (Table 3). This mismatched between SIME and Lassen plot estimates has been already shown by Schain and colleagues [35], who reported similar *V*_ND_ values for both healthy controls (mean±sd: 1.45 ± 0.56 mL/cm^3^) and Alzheimer disease patients (mean±sd: 1.36 ± 0.40 mL/cm^3^). In a recent study, comparing SIME *V*_ND_ estimates across different populations, Laurell and colleagues found a mean value of 1.12 mL/cm^3^ for a group of 6 HABs first-episode psychosis patients, very well aligned with the SIME *V*_ND_ estimate obtained in our sample [25].

**Table 3:** Individual *V*_ND_ estimates with SIME method and with Lassen plot after XBD173 blocking

	Standard 2TCM modeling			2TCM1K modeling
Subject ID	SIME *V*_ND_ from baseline scan (mL/cm^3^)	Lassend plot *V*_ND_ (mL/cm^3^) [95% CI]	Rel Dif (%)	Lassend plot *V*_ND_ (mL/cm^3^) [95% CI]
#305	1.24	5.55 [4.46, 6.43]	−78%	0.14 [0.11, 0.16]
#314	0.94	3.05 [2.45, 3.53]	−69%	0.78 [0.63, 0.91]
#316	1.68	2.31 [1.86, 2.67]	−27%	1.03 [0.83,1.19]
#337	1.14	1.51 [1.17, 1.78]	−24%	0.27 [0.21, 0.32]
#338	1.17	2.09 [1.68, 2.41]	−44%	1.01 [0.81, 1.16]
#339	0.71	1.67 [1.34, 1.94]	−58%	0.89 [0.71, 1.03]
#378	1.24	1.50 [1.20, 1.73]	−17%	0.75 [0.60, 0.86]

## Discussion

To our knowledge, this is the first PET study to pharmacologically estimate the non-displaceable binding component (*V*_ND_) in patients with schizophrenia. We find that the *V*_ND_ of [^11^C]PBR28 in schizophrenia to be 1.99 mL/cm^3^, a value that is very similar to the one previously reported for healthy controls (1.98 mL/cm^3^) [31]. We also find that a substantial component of the [^11^C]PBR28 PET signal is displaced by XBD173 in schizophrenia, indicating specific binding to TSPO.

Before we discuss the implications of this finding, we should address the potential limitations of this study. First, only one patient included in this study was unmedicated, with the remaining patients medicated with antipsychotic medication. The relationship between antipsychotic exposure and expression levels of the translocator protein (TSPO) remains unclear, although a rat study found no effect of antipsychotics on PBR28 (Bloomfield et al. 2018). Second, we have only scanned patients who were high affinity binders for TSPO. Therefore, in order for our results to be generalized, we need to assume that *V*_ND_ is consistent across binding affinity groups. Importantly, genotype polymorphisms are supposed to only affect the affinity of the tracer to TSPO, and therefore no differences in *V*_ND_ are thought to exist between genotype groups. This is supported by in vitro data using 3H-PBR28, which showed that *V*_ND_ does not differ across binding affinity class [30]. However, some data suggest that there may be a higher ratio of specific to non-displaceable binding in HABs compared with MABs [23, 31]. In a previous study using XBD173 to determine *V*_ND_ in healthy controls, BP_ND_ was higher in HABs compared with MABs in all ROIs studied [31]. However, the authors used a polymorphism plot to calculate an independent population estimate of *V*_ND_ which produced very similar population *V*_ND_ estimates (1.89 to 2.00). Future studies should aim to independently determine if *V*_ND_ is different among different binding affinity subjects. Third, we scanned subjects separated by an average of almost 2-year interval. It has been suggested that TSPO expression may change during the course of the disorder, but a recent study using BP as an outcome measure showed no differences between at-risk mental state individuals, recent onset schizophrenia, and chronic schizophrenia [10]. Furthermore, one of our study participants had the baseline and follow-up PET scan with 1-week interval and there was no difference in *V*_T_ or *V*_ND_ between this patient and the remaining ones. Finally, some methodological considerations should be taken into account. The occupancy plot is a simple and very common method to compute the tracer displaceable binding but it is not without limitation [27, 36]. First of all, there are alternative formulations to calculate the fractional receptor availability in molecular imaging studies [14, 24]. Moreover, the occupancy plot is based on ordinary least squares that assumes noise-free independent variables and no autocorrelated measurement error. But this is not necessarily the case for the occupancy plot variables (both baseline *V*_T_ and delta *V*_T_ are noisy measures and regional estimates tend to be correlated with each other) and both the noise level and the variability of measurements can change depending on the radioligand and its target as well as on the set of ROIs used for the regression. Alternative estimation methods, which take into account noise on both axes, may be considered (e.g., likelihood estimation [28] or orthogonal regression) [42]). These methods, similarly to weighted estimation approaches, necessitate assumptions about the noise distributions of the measurements. To compare the results of this work with existent literature, we consistently use the same approach reported by other occupancy studies with PBR28 PET and XBD173. However, further investigations are needed to test the sensitivity of PBR28 *V*_ND_ estimates to the quantification methodology. Also the use of individual *V*_ND_ estimates requires further consideration. The individual *V*_ND_ estimates obtained for our subjects ranged between 1.50 and 5.55 for 2TMC, between 0.14 and 1.03 for 2TMC1K, and between 0.71 and 1.24 for the SIME method. From this data alone, it is not possible to ascertain whether the inter-subject variability represents true biological variability or is simply a consequence of the increased noise obtained from unconstrained fits. On the contrary, constraining the *V*_ND_ of all the subjects to the same population value is a very strong assumption, which opportunistically has the advantage of reducing the inter-subject variability. However, irrespective of the quantification method used, the reduction of tracer binding after XBD173 administration was global, supporting the idea TSPO is obviously distributed and that no brain region could be used as a true reference region with no or negligible tracer-specific binding. This implies to control for the variation of tracer-specific signal when a particular region is used as normative (or pseudo reference) region for the quantification of tracer binding across different groups of participants or before and after interventions that could modify brain TSPO expression [1].

Our finding that *V*_ND_ is not altered in schizophrenia has implications for the interpretation of TSPO PET studies in schizophrenia. It shows that differences between groups are unlikely to be due to differences in *V*_ND_, indicating that where *V*_T_ differences are reported they most likely represent alterations in specific binding in blood or brain. It also indicates that lower non-specific binding does not explain the discrepant findings between studies reporting BP_ND_ and those reporting *V*_T_ ([26]; Plavén-Sigray P et al., 2018). The XBD173 binds to the translocator protein (18 kDa) with nanomolar affinity and shows negligible affinity to a broad range of neurotransmitter receptors (Kita et al., 2004) and our finding indicates a large component of PET signal in schizophrenia is therefore specific binding to TSPO.

## Conclusions

In conclusion, we have used a pharmacological blockade of TSPO with XBD173 to calculate a population level [^11^C]PBR28 *V*_ND_. Our results show a substantial component of the [^11^C]PBR28 signal is specific binding to TSPO specific in schizophrenia patients and that the *V*_ND_ in patients with schizophrenia is similar to that previously reported in healthy controls, indicating that altered non-specific binding does not account for discrepant PET findings in schizophrenia.

## Supplementary information

Fig S1 (DOCX 1069 kb)

Fig S2 (DOCX 1044 kb)

Fig S3 (DOCX 1049 kb)
