# Supplementary material for: Specific and non-specific binding of a tracer for the translocator-specific protein in schizophrenia: an [11C]-PBR28 blocking study
Source: Eur J Nucl Med Mol Imaging. 2021 Apr 6;48(11):3530–9. doi: 10.1007/s00259-021-05327-x (PMC8440284; doi:10.1007/s00259-021-05327-x)
Supplement: Supplementary file 1 — (DOCX 1069 kb) [file 259_2021_5327_MOESM1_ESM.docx]

*Figure S1 - Logan VT parametric mapping before and after XBD173 administration in a representative subject.*

*
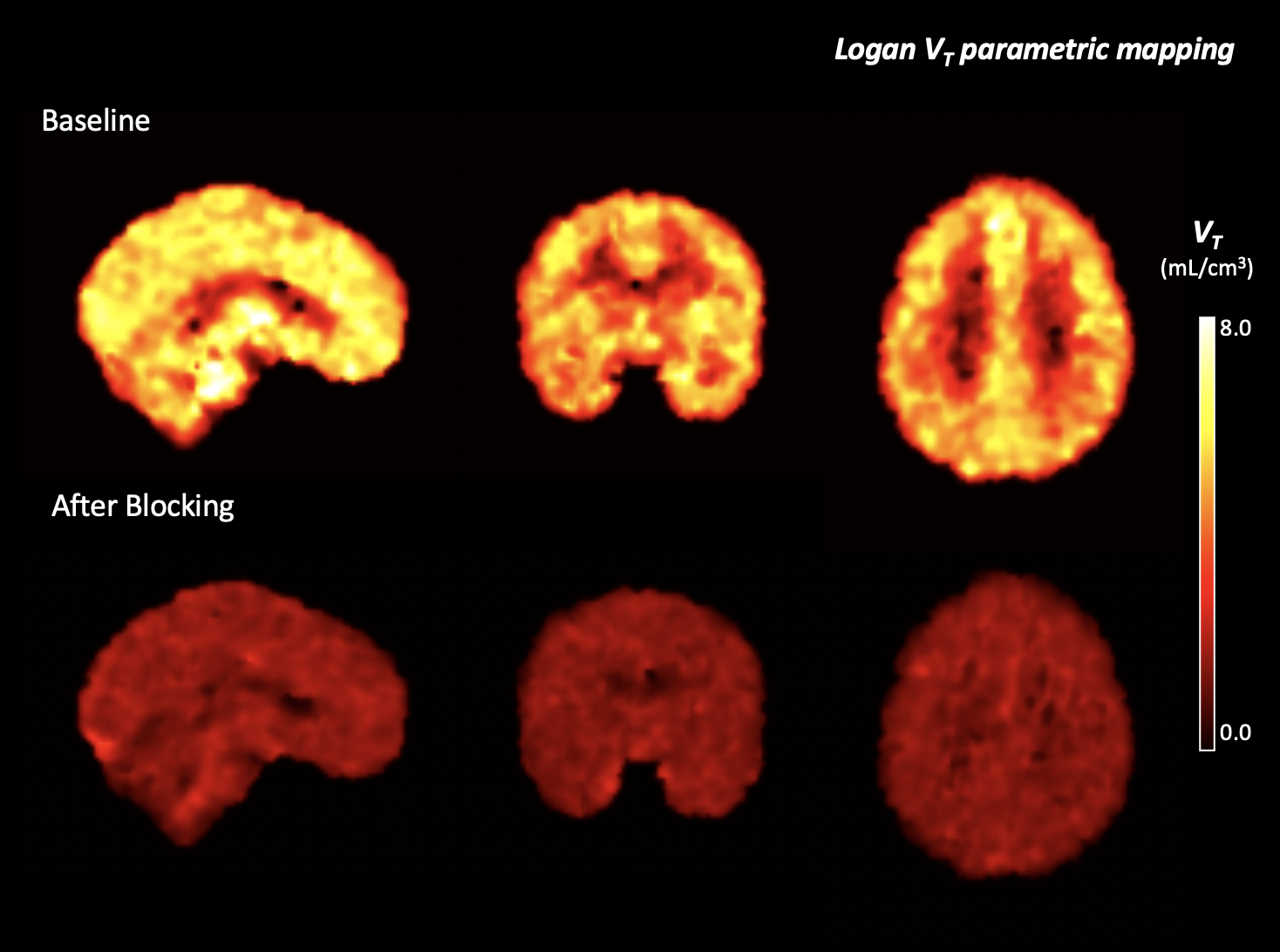
*

*Ref:* [*https://journals.sagepub.com/doi/10.1038/jcbfm.1990.127*](https://journals.sagepub.com/doi/10.1038/jcbfm.1990.127)
