# Supplementary material for: Specific and non-specific binding of a tracer for the translocator-specific protein in schizophrenia: an [11C]-PBR28 blocking study
Source: Eur J Nucl Med Mol Imaging. 2021 Apr 6;48(11):3530–9. doi: 10.1007/s00259-021-05327-x (PMC8440284; doi:10.1007/s00259-021-05327-x)
Supplement: Supplementary file 2 — (DOCX 1044 kb) [file 259_2021_5327_MOESM2_ESM.docx]

*Figure S2 - VT parametric mapping solved with 2TCM1K and variational bayes regression before and after XBD173 administration in a representative subject.*

*
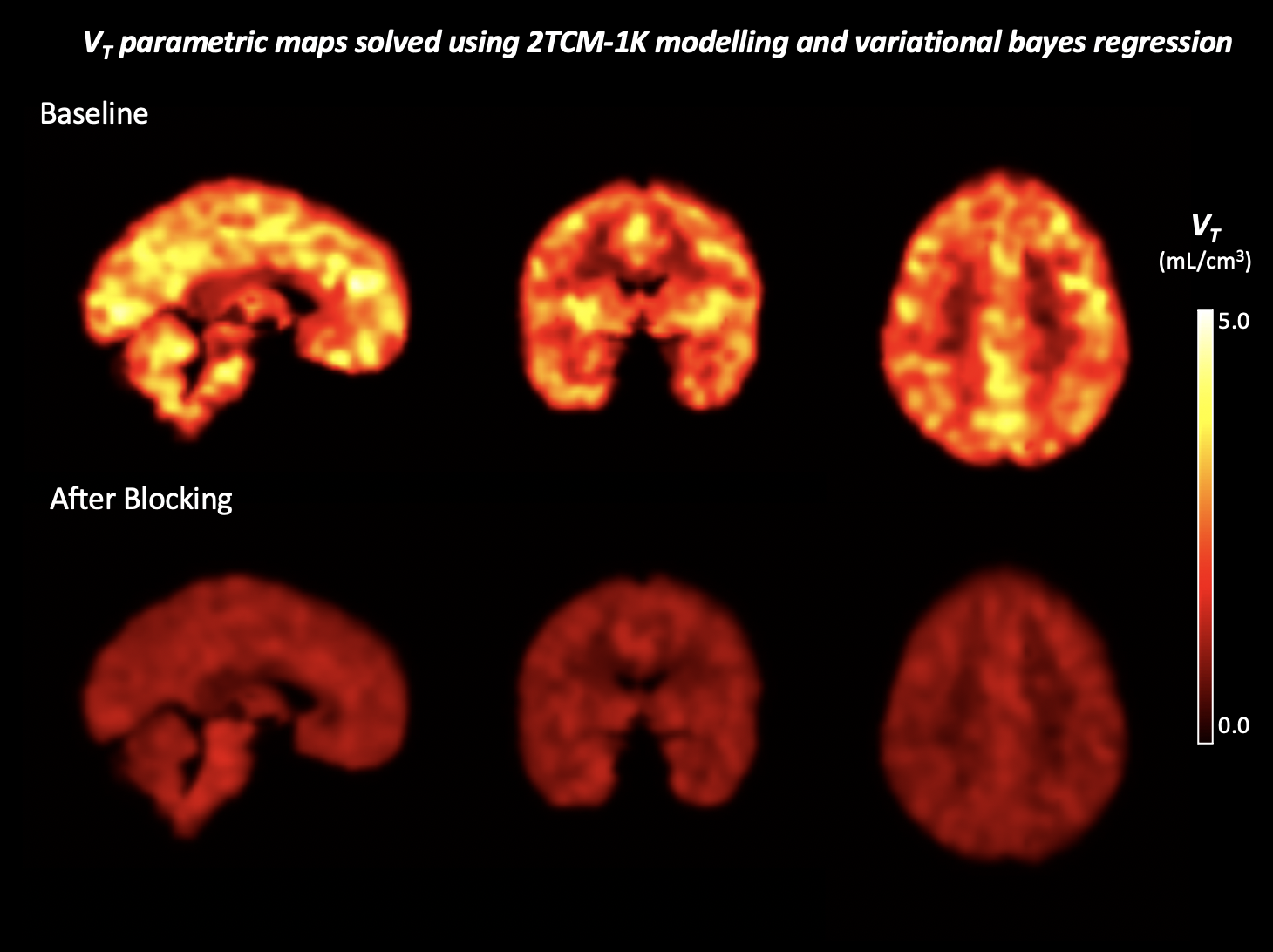
*

*Ref:* <https://doi.org/10.1016/j.neuroimage.2017.02.009>
