# Supplementary material for: Specific and non-specific binding of a tracer for the translocator-specific protein in schizophrenia: an [11C]-PBR28 blocking study
Source: Eur J Nucl Med Mol Imaging. 2021 Apr 6;48(11):3530–9. doi: 10.1007/s00259-021-05327-x (PMC8440284; doi:10.1007/s00259-021-05327-x)
Supplement: Supplementary file 3 — (DOCX 1049 kb) [file 259_2021_5327_MOESM3_ESM.docx]

*Figure S3 - Spectral Analysis Impulsive Response Function parametric mapping before and after XBD173 administration in a representative subject quantified at 90 minutes after injection.*

*
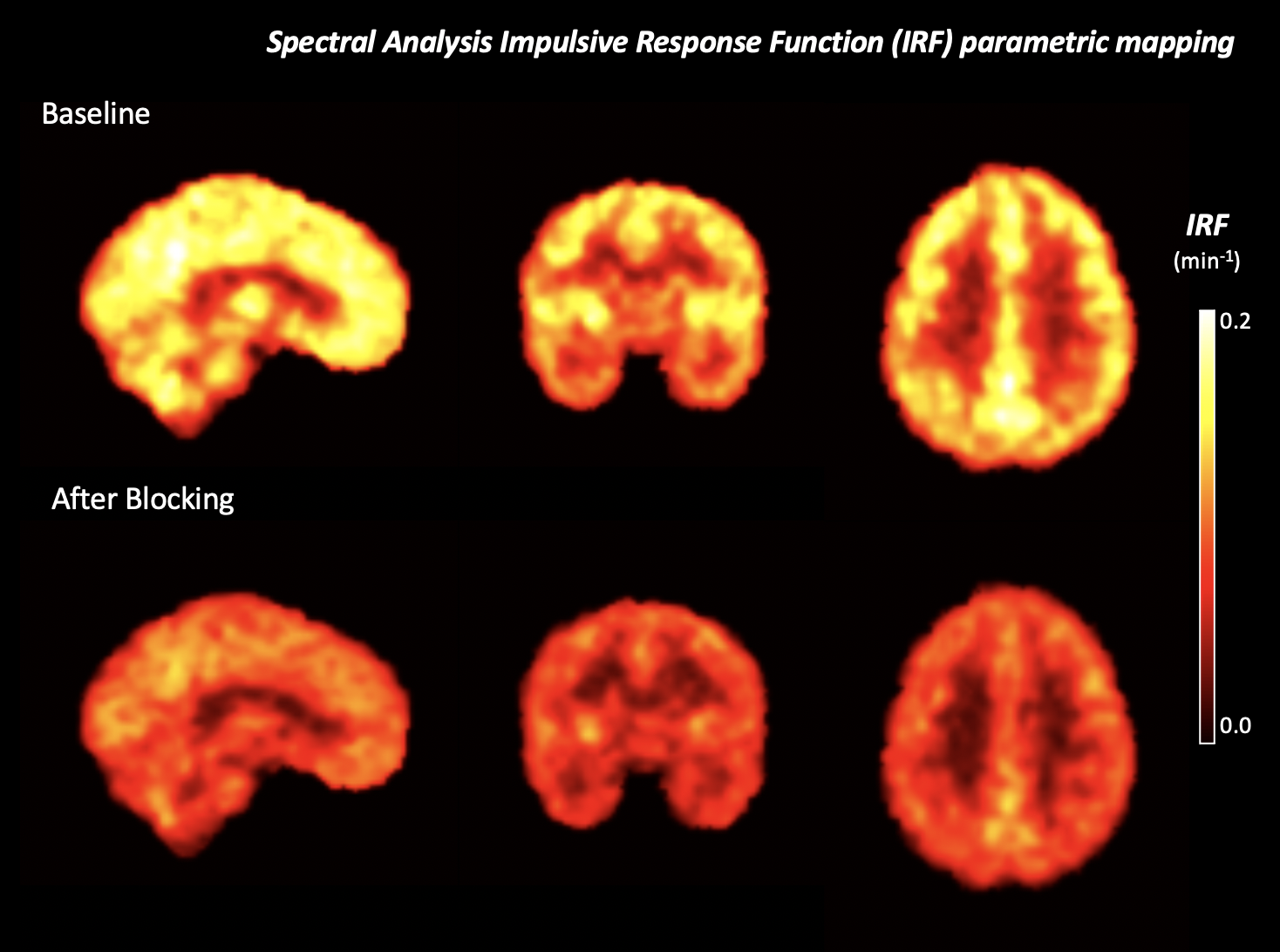
*

*Ref:* [*https://pubmed.ncbi.nlm.nih.gov/29523926/*](https://pubmed.ncbi.nlm.nih.gov/29523926/)
